# A Cross-Sectional Study on the Estimation of Urine Albumin for the Early Diagnosis of Diabetic Nephropathy Among Patients With Diabetes Mellitus at a Tertiary Care Hospital in Central India

**DOI:** 10.7759/cureus.45522

**Published:** 2023-09-19

**Authors:** Saleha S Ansari, Dr. Ranjana Sharma

**Affiliations:** 1 Medical Surgical Nursing, Smt. Radhikabai Meghe Memorial College of Nursing, Datta Meghe Institute of Higher Education & Research, Wardha, IND

**Keywords:** diet patterns, diabetes duration, family history, demographics, early diagnosis, albuminuria, diabetic nephropathy

## Abstract

Background

Diabetic nephropathy is a significant concern among individuals with diabetes mellitus, warranting early diagnosis for effective management. This study focuses on the potential of urine albumin estimation as an early diagnostic tool for diabetic nephropathy among patients in central India.

Methods

A cross-sectional methodology involved 65 individuals diagnosed with diabetes mellitus at a tertiary care hospital. Demographic factors, including age, gender, family history, duration of diabetes, and dietary patterns, were gathered. Urine albumin levels were categorized as “normal,” “microalbuminuria,” and “macroalbuminuria.” The collected data were analyzed using IBM SPSS Statistics, version 24.0 (IBM Corp., Armonk, NY). Qualitative variables were presented as percentages and counts. The comparison between groups was conducted using the chi-square exact test. Quantitative variables were described as mean (±standard deviation) and median.

Results

The study reveals that 78.5% of cases exhibited normal levels below 30 mg, with a mean value of 1.00 ± 0.414. Microalbuminuria, characterized by 30-300 mg levels, was observed in 21.5% of cases. Importantly, no instances of macroalbuminuria, with levels exceeding 300 mg, were detected among the participants. There are associations between demographic variables and diabetic nephropathy findings. Age and gender displayed non-significant associations. Family history of diseases, particularly diabetes, showed significance. Diabetes duration demonstrated a significant link, while diet patterns displayed no significant associations.

Conclusion

This study contributes insights into the complex interactions of demographic factors in diabetic nephropathy. Early identification and intervention, guided by the associations observed, could enhance patient outcomes and mitigate the burden of diabetic nephropathy-related complications. Further research is warranted to validate and extend these findings to diverse populations.

## Introduction

Diabetic nephropathy is a progressive renal ailment resulting from diabetes mellitus, a metabolic disorder characterized by elevated blood sugar levels [1]. This particular kidney affliction has escalated into a pressing global concern within public health [2]. Notably, it assumes a significant role as a leading cause of morbidity and mortality among individuals grappling with diabetes. The intertwined relationship between diabetes and kidney dysfunction necessitates the utmost attention to curtail the adverse impact of this complication [3].

In this intricate interplay, timely detection and proactive medical interventions manifest as pivotal factors. The trajectory of diabetic nephropathy is marked by a gradual advancement, often evolving insidiously and thereby underscoring the urgency of early diagnosis. Swift and accurate intervention holds the promise of curbing the progression of diabetic nephropathy, thereby mitigating the dire consequences it entails [4]. The imperative lies in not only arresting the advancement of this renal ailment but also circumventing the myriad complications it associates with, such as cardiovascular ailments and renal failure [5].

Among the array of biomarkers, the prominence of urinary albumin levels emerges as a noteworthy signifier of early-stage renal dysfunction among diabetic patients. Albumin, a protein in the blood, may be excreted in the urine when the kidney’s filtration barrier becomes compromised [6]. This phenomenon, termed “microalbuminuria,” is a sentinel signal indicative of the onset of kidney damage. Consequently, accurately quantifying urine albumin levels has garnered substantial attention as a diagnostic instrument. This tool not only holds the potential to facilitate the identification of diabetic nephropathy at its nascent juncture but also augments the prognostic aspect of patient management [7].

In this context, cross-sectional studies are crucial in investigating the prevalence and patterns of diseases within a specific population at a given time. This study aims to contribute to understanding the early detection of diabetic nephropathy by assessing the utility of urine albumin estimation among patients with diabetes mellitus. The research is conducted in a tertiary care hospital in central India, where diabetes is notably high.

This study’s importance lies in its potential for improving the clinical management and outcomes of diabetic nephropathy. By identifying individuals at an early stage of kidney dysfunction, healthcare providers can initiate interventions to prevent or delay the progression of the disease. This could ultimately reduce the burden of end-stage renal disease and improve the overall quality of life for individuals with diabetes.

## Materials and methods

Study setting and design

The cross-sectional study was conducted at AVBR Hospital Sawangi in the Wardha district in March 2022. The study focused on patients with type 2 diabetes mellitus.

Study population

This study employed a purposive sampling technique to recruit type 2 diabetic mellitus patients. A total of 65 patients diagnosed with type 2 diabetes mellitus were selected. The inclusion criteria comprised male and female patients aged eight to 15 years after being diagnosed with type 2 diabetes mellitus.

Sample selection criteria

Inclusion Criteria

During the process of selecting samples for our study, we established specific inclusion criteria to ensure the relevance and suitability of participants. First and foremost, we were looking for individuals who had been officially diagnosed with type 2 diabetes mellitus. This diagnosis served as a fundamental requirement for eligibility in our study. Furthermore, we were open to both male and female patients, as we aimed to gather data that were representative of the broader population. Additionally, we were interested in patients who had been living with type 2 diabetes mellitus for a period spanning from eight to 15 years.

Exclusion Criteria

Equally important in our sample selection process were the exclusion criteria, which helped us identify individuals who should not participate in the study. Specifically, we did not include patients who had already been diagnosed with diabetic nephropathy or any other kidney diseases. This exclusion was essential to maintain the focus of our research on the aspects directly related to type 2 diabetes mellitus. Additionally, we did not involve patients who were unwilling to participate, as voluntary consent was a cornerstone of ethical research practices. By adhering to these criteria, we aimed to ensure the integrity and reliability of our study’s results.

Data collection

Collecting sufficient and relevant data is a pivotal aspect of any research endeavor, aiding in addressing the study’s fundamental question: the early diagnosis of diabetic nephropathy among diabetes mellitus patients through urine albumin estimation. Detailed urine samples were procured for a thorough examination. A positive rapport was maintained throughout the process. On the initial day, urine samples were collected from participants, who were also asked to provide demographic information such as age, gender, family medical history, duration of diabetes mellitus, and dietary patterns. Urine estimation was conducted on the same day.

Statistical analysis

The gathered data underwent analysis using IBM SPSS Statistics, version 24.0 (IBM Corp., Armonk, NY). Demographic data were acquired through a questionnaire specifically developed by the researchers. Mean scores were computed and evaluated. Chi-square analysis was utilized to ascertain the connection between the dependent variable (type 2 diabetes mellitus) and the independent variables (socio-demographic profile). A statistical significance level was considered achieved if the p-value was less than 0.05, maintaining a confidence level of 95%.

Sample collection and laboratory analysis

Urine samples were collected from the study participants and stored in sterile screw-top containers. These samples were then carefully sealed in airtight containers and transported in sealed bags to the laboratory. Upon arrival, the urine albumin test was carried out, and the urine albumin (protein) levels were measured using the urine dipstick method [8].

Ethical considerations

Every participant was required to provide written informed consent after receiving a comprehensive explanation of the study’s concepts and objectives. Participants were assured of their privacy and the confidentiality of their information. DMIMS(DU)/IEC/Dec-2021/284 thoroughly reviewed and approved the study protocol.

## Results

Table 1 outlines the demographic characteristics of the study participants, offering a snapshot of key variables. Age distribution revealed that 35.4% fell in the 41-50 age group, while 50.8% were aged 51-60, and 13.8% were between 61-70 years. Based on gender, 56.9% were male and 43.1% were female. Family medical history indicated that 40% had a history of diabetes, 26.2% of hypertension, 3.1% of chronic kidney disease (CKD), 4.6% of heart disease, and 26.2% had no history of these ailments. Regarding diabetes duration, 30.8% were diagnosed eight to 10 years ago; 44.6%, 11-12 years ago; 16.9%, 13-15 years ago; and 7.7%, over 15 years ago. Diet patterns comprised 18.5% of vegetarians and 81.5% following a mixed diet.

**Table 1:** Demographic characteristics of the study population CKD, chronic kidney disease

Demographic variable	Frequency	Percentage
Age		
41-50 years	23	35.4%
51-60 years	33	50.8%
61-70 years	9	13.8%
Gender		
Male	37	56.9%
Female	28	43.1%
Family history of the following disease		
Diabetes	26	40%
Hypertension	17	26.2%
CKD	2	3.1%
Heart disease	3	4.6%
No any history of the disease	17	26.2%
Since how many years have you been diagnosed with diabetes mellitus		
8-10 years	20	30.8%
11-12 years	29	44.6%
13-15 years	11	16.9%
More than 15 years	5	7.7%
Diet pattern		
Vegetarian	12	18.5%
Mix-diet	53	81.5%

Table 2 illustrates the outcomes of urine albumin assessments conducted to identify the early stages of diabetic nephropathy among diabetes mellitus patients. The scores were categorized as “normal,” with urine albumin levels below 30 mg, constituting 78.5% of cases, and “microalbuminuria,” spanning 30-300 mg, encompassing 21.5% of cases. Notably, no instances of “macroalbuminuria,” characterized by urine albumin levels exceeding 300 mg, were observed among the participants.

**Table 2:** Urine albumin for early diagnosis of diabetic nephropathy among diabetes mellitus patients

Score range		Urine albumin		Mean ± SD
		Frequency	Percentage	
Normal	<30 mg	51	78.5%	1.00 ± 0.414
Microalbuminuria	30-300 mg	14	21.5%	

Table 3 presented associations related to diabetic nephropathy findings across different demographic variables. Divided into “normal” and “microalbuminuria” groups with respective frequencies, the table presents chi-square values, degrees of freedom, and p-values as measures of association. Regarding age, gender, and diet pattern, no significant associations were found.

**Table 3:** Association with the findings of diabetic nephropathy NS, non-significant; S, significant; CKD, chronic kidney disease

Demographic variable		Normal	Microalbuminuria	Chi-square value	df	p-value
Age	41-50 years	19	4	3.248	2	0.197 NS
		82.6%	17.4%			
	51-60 years	27	6			
		81.8%	18.2%			
	61-70 years	5	4			
		55.6%	44.4%			
Gender	Male	28	9	0.394	1	0.530 NS
		75.7%	24.3%			
	Female	23	5			
		82.1%	17.9%			
Family history of the following disease	Diabetes	24	2	12.306	4	0.015 S
		92.3%	7.7%			
	Hypertension	12	5			
		70.6%	29.4%			
	CKD	0	2			
		0.0%	100.0%			
	Heart disease	3	0			
		100.0%	0.0%			
	No any history of the disease	12	5			
		70.6%	29.4%			
Since how many years were you diagnosed with diabetes mellitus	8-10 years	19	1	23.911	3	0.001 S
		95.0%	5.0%			
	11-12 years	25	4			
		86.2%	13.8%			
	13-15 years	7	4			
		63.6%	36.4%			
	More than 15 years	0	5			
		0.0%	100.0%			
Diet pattern	Vegetarian	9	3	0.104	1	0.747 NS
		75.0%	25.0%			
	Mix-diet	42	11			
		79.2%	20.8%			

## Discussion

The findings of this cross-sectional study provide valuable insights into the early diagnosis of diabetic nephropathy among patients with diabetes mellitus. The study’s primary focus on assessing the utility of urine albumin levels as a diagnostic tool for identifying kidney dysfunction in its incipient stages contributes to the existing body of knowledge in this field. The study’s demographic analysis and associations with albuminuria status shed light on the complex interplay between various factors and the development of diabetic nephropathy.

The lack of significant associations between age and gender with albuminuria status aligns with previous research indicating that diabetic nephropathy can affect individuals across a wide age range and genders without particular predispositions [9]. This underscores the importance of monitoring kidney function universally among diabetes patients, regardless of age or gender.

The noteworthy correlation identified between a family history of CKD and heart disease with albuminuria status is a captivating discovery. Recognizing family history as a long-standing risk factor for various chronic ailments, including kidney dysfunction, underscores the significance of this finding [10]. Among the pivotal risk factors contributing to microalbuminuria, elevated blood pressure holds a prime position. The mechanism through which high blood pressure could induce microalbuminuria involves increased glomerular filtration pressure, subsequently leading to renal damage [11]. This observation underscores the genetic influence in the progression of diabetic nephropathy and underscores the necessity for targeted interventions in patients with such familial backgrounds.

The association between the duration since diabetes diagnosis and albuminuria status aligns with the established understanding that a lengthier duration of diabetes is linked to an elevated risk of kidney complications [12]. Prolonged hyperglycemia can induce structural and functional kidney alterations, thus emphasizing the importance of regular monitoring and timely intervention. Furthermore, several other risk factors contribute to the escalation of albuminuria to more severe levels, including higher baseline levels of albuminuria, poorer glycemic control as reflected by HbA1c levels, increased blood pressure, and cigarette smoking [13-15].

Excessive salt consumption leads to an augmented volume load, consequently heightening intra-glomerular pressure and glomerular hyperfiltration. This, in turn, leads to the excretion of albumin in the urine. Additionally, an elevation in blood pressure plays a role in the mechanism behind salt-induced albuminuria. An elevated fasting plasma glucose level indicates underlying insulin resistance, which promotes sodium re-absorption and could contribute to the volume load. Alternatively, endothelial dysfunction might explain the observed link between urinary albumin excretion and other contributing factors [16,17].

The study’s findings also affirm the importance of urine albumin assessment in early diabetic nephropathy diagnosis. The prevalence of microalbuminuria suggests that subtle kidney dysfunction is present even in patients without overt symptoms. This underscores the potential of albuminuria as a sensitive marker for identifying kidney impairment before it progresses to advanced stages.

It is worth noting that diet patterns did not show a significant association with albuminuria status in this study. While dietary factors can influence kidney health, other variables might have contributed to the lack of observed association. Future studies could explore this aspect further, considering broader dietary patterns and their potential impact on kidney function.

Limitation

This study possesses several limitations that influence the interpretation of its findings. The cross-sectional design restricts the ability to establish causality, necessitating longitudinal investigations for a clearer understanding. Conducted within a single tertiary care hospital in central India, the generalizability of results to broader populations might be constrained due to regional variations. Reliance on self-reported family history and diet patterns introduces potential recall bias, while not accounting for other relevant biomarkers and kidney function aspects may limit the study’s comprehensiveness.

## Conclusions

In conclusion, this study sheds light on the crucial role of urine albumin estimation as an early diagnostic tool for diabetic nephropathy within the context of diabetes mellitus patients at a central India tertiary care hospital. The findings underline the significance of timely detection and intervention to mitigate the impact of diabetic nephropathy, a significant global health concern. Notably, the associations between a family history of CKD and heart disease with albuminuria status highlight the genetic component in the development of kidney dysfunction. Additionally, the duration since diabetes diagnosis emerges as a significant predictor emphasizes the importance of continuous kidney function monitoring. Although diet patterns did not display significant associations, urine albumin levels prove promising as indicators of early-stage diabetic nephropathy. This research advances the comprehension of demographic factors’ interplay with diabetic nephropathy, emphasizing the critical need for proactive measures to enhance patient outcomes and reduce the burden of this complication.
